# The optimal number of initial prostate biopsy cores in daily practice: a prospective study using the Nara Urological Research and Treatment Group nomogram

**DOI:** 10.1186/s13104-015-1668-9

**Published:** 2015-11-18

**Authors:** Nobumichi Tanaka, Keiji Shimada, Yoshinori Nakagawa, Shuya Hirao, Shuji Watanabe, Makito Miyake, Satoshi Anai, Akihide Hirayama, Noboru Konishi, Kiyohide Fujimoto

**Affiliations:** Department of Urology, Nara Medical University, 840 Shijo-cho, Kashihara, Nara, 634-8522 Japan; Department of Pathology, Nara Medical University, Kashihara, Nara, Japan; Nara Urological Research and Treatment Group, Kashihara, Nara, Japan; Department of Urology, Nara Hospital Kinki University Faculty of Medicine, Ikoma, Nara, Japan

**Keywords:** Prostate cancer, Prostate biopsy, Prostate volume, Volume biopsy ratio, Prospective study

## Abstract

**Background:**

To elucidate the optimal number of prostate biopsy cores using a nomogram allocating 6–12 biopsy cores, the number generally used in daily practice, based on age and prostate volume (PV).

**Methods:**

We enrolled 936 patients who received an initial prostate biopsy from April 2006 to January 2009. A number of 6–12 biopsy cores was allocated based on age and PV Nara Urological Research and Treatment Group (NURTG) nomogram. To elucidate the predictive parameters of cancer detection in patients with a prostate specific antigen (PSA) value in the gray zone, univariate and multivariate logistic regression analysis were carried out.

**Results:**

The total cancer detection rate and the cancer detection rate in the PSA gray zone (4.1–10.0 ng/mL) were 48.0 and 37.6 %, respectively. The cancer detection rates in the gray zone stratified by patient age of ≤59, 60–64, 65–69, 70–74, 75–79, and ≥80 years were 28.4, 35.0, 26.9, 37.9, 45.7, and 54.8 %, respectively. The significant predictive parameters of cancer detection in the gray zone were age, volume biopsy ratio (VBR: PV divided by number of biopsy cores), PSA density (PSAD), digital rectal examination findings, and transrectal ultrasound findings in univariate analyses. Finally, age, VBR, and PSAD were independent parameters to predict cancer detection in the gray zone. The adverse event profile was acceptable.

**Conclusions:**

Our present study revealed that the cancer detection rate using the NURTG nomogram allocating 6–12 biopsy cores, the number generally used in daily practice, based on age and PV, could provide similar efficacy as previous studies involving more biopsy cores. In older patients the number of biopsy cores could be reduced.

## Background

Since the concept of systematic prostate biopsy has been introduced, the number of biopsy cores has increased to 12 cores. At first, sextant systematic biopsy was proposed [1]. The number of cores gradually increased to improve the cancer detection rate. Remzi et al. introduced the Vienna nomogram, allocating a number of 6–18 (mean 10) cores based on the patients’ age and prostate volume (PV). They concluded that the cancer detection rate was 36.7 % in the gray zone of prostate specific antigen (PSA) (4.0–10.0 ng/mL) [2]. The concept of Remzi’s report to decide the number of cores based on age and PV is appropriate, because the meaning of the same number of cores for prostates of different volume is unscientific. The volume guaranteed by a biopsy core varied. For example, if 12 core biopsies were performed in patients with a 60 mL prostate, one core would cover 5 mL, but in case of a 12 mL prostate one core would cover 1 mL. We believe that the number of cores should be determined based on PV. To increase the number of cores increases the cancer detection rate. On the other hand, the optimal number of cores is an open question.

To shed light on these issues, we conducted this prospective study to evaluate the NURTG (Nara Urological Research and Treatment Group: former Nara Urological Oncology Research Group: NUORG) nomogram allocating 6–12 biopsy cores, the number generally used in daily practice, based on age and PV.

## Methods

This prospective study was conducted at Nara Medical University and its three affiliate hospitals (Yamatotakada City Hospital, Hirao Hospital, and Saiseikai Chuwa Hospital). We enrolled 936 patients who received an initial prostate biopsy from April 2006 to January 2009. The criteria for the indication of biopsy were abnormal PSA value, abnormal findings of digital rectal examination (DRE) and abnormal findings by transrectal ultrasound. Patients were excluded from this study if they had an acute or chronic prostatitis, urinary retention, urinary tract infection or indwelling urinary catheter.

**Fig. 1:**
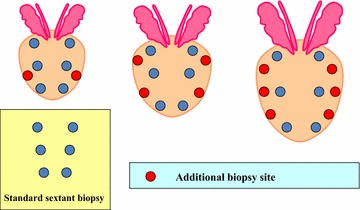
The principal portion and additional portion of biopsy cores. *Red circle* indicates additional site and *blue circle* indicates standard sextant systematic biopsy

We adopted the age-specific reference range of PSA for the cut-off value of PSA [3]. The PSA cut-off value were 3.1 ng/mL for patients less than 65 years, 3.6 ng/mL for 65–69 years, and 4.1 ng/mL for 70 years or older, respectively. The number of biopsy cores allocated was from 6 to 12 cores based on age and PV (NURTG nomogram) (Table 1). This nomogram was designed based on the previous reports by Remzi [2] and Ito [4]. The maximum number of biopsy cores were 18 [2] and 20 [4] in the previous studies. In the present study, we set the maximum number of biopsy cores as 12 cores because 12 cores is an appropriate number in daily practice. The principal portion of the biopsy was that of sextant systematic biopsy. Additional cores were located to the far lateral region (Fig. 1). Prostate needle biopsy was performed using an automatic biopsy gun with an 18-gauge needle under transrectal ultrasound (TRUS) guidance. PV was calculated using the formula for prostate ellipsoid (transverse width × transverse length × longitudinal height × 0.52). Two pathologists (K.N. and S.K.), both experts in prostate cancer diagnosis, centrally reviewed the Gleason score of all biopsy specimens.

**Table 1:** The number of biopsy cores stratified by age and prostate volume (NURTG nomogram)

Prostate volume (mL)	Age			
	≤59	60–64	65–69	70≤
−25	12	10	8	6
25–50	12	12	10	8
50−	12	12	12	10

We evaluated the cancer detection rate of the NURTG nomogram in all patients as well as patients with a PSA value in the gray zone (4.1–10.0 ng/mL). The statistical difference between the cancer group and the non-cancer group for categorical variables was tested by the Chi-square test, while that for continuous variables was tested by the t-test. We evaluated adverse events of grade 2 or greater related prostate biopsy using common terminology criteria adverse events version 4.0. To elucidate the predictive parameters of cancer detection in patients with a PSA value in the gray zone, univariate and multivariate logistic regression analysis were carried out using clinical parameters such as age, volume biopsy ratio (VBR: PV divided by number of cores), PSA density (PSAD: PSA value divided by PV) [5], DRE findings, and TRUS findings. Variables that were found to be significant in the univariate analyses (p < 0.05) were entered into the multivariate analysis. All statistical analyses were performed using PASW statistics 17.0 (SPSS Inc., Chicago, IL, USA). All p values of less than 0.05 were considered statistically significant.

The institutional reviewer board (Nara Medical University) approved this prospective study, and informed consent by the patients was exempted in view of the aim and methods of this study.

## Results

The patient characteristics are shown in Table 2. The median age and PSA value were 70 years and 5.8 ng/mL, respectively, in the no cancer group, and 73 years and 10.1 ng/mL, respectively in the cancer group. The median age and PSA value of the cancer group were significantly higher than those in the no cancer group (p < 0.001, and p = 0.008). The median PV and PSAD were 38.8 mL and 0.15 ng/mL/mL, respectively, in the no cancer group, and 28.1 mL and 0.41 ng/mL/mL, respectively, in the cancer group. The median PV in the no cancer group was significantly larger than that in the cancer group (p < 0.001), while the median PSAD in the no cancer group was significantly smaller than that in the cancer group (p = 0.005). The median VBR of the no cancer group was 4.22, while that in the cancer group was 3.64. The median VBR of the no cancer group was significantly larger than that in the cancer group (p < 0.001). The distribution of a Gleason score of ≤6, 7, and 8–10 were 32.0, 47.2 and 20.8 %, respectively.

**Table 2:** Patients’ background

	All patients (n = 936)	No cancer (n = 487)	Cancer (n = 449)	p value
Age				<0.001
Mean (median)	71 (71)	69 (70)	73 (73)	
Range	22–94	22–89	49–94	
PSA (ng/mL)				0.008
Mean (median)	58.5 (7.1)	7.4 (5.8)	114.0 (10.1)	
Range	0.3–16,920	0.3–83.6	0.6–16,920	
PV (mL)				<0.001
Mean (median)	38.0 (33.2)	43.6 (38.8)	32.0 (28.1)	
Range	6–176	6–176	10–155	
PSAD (ng/mL/mL)				0.005
Mean (median)	1.99 (0.22)	0.21 (0.15)	3.94 (0.41)	
Range	0.0–480.7	0.0–3.1	0.0–480.7	
VBR (volume biopsy ratio) (mL/core)				<0.001
Mean (median)	4.17 (3.89)	4.50 (4.22)	3.80 (3.64)	
Range	0.46–17.62	0.46–17.62	0.86–15.47	
Number of biopsy cores				<0.001
6	169	41 (24.3)	128 (75.7)	
8	331	161 (48.6)	170 (51.4)	
10	237	130 (54.9)	107 (45.1)	
12	199	155 (77.9)	44 (22.1)	
Gleason score, no (%)				
≦6	143		143 (32.0)	
7	211		211 (47.2)	
8–10	93		93 (20.8)	

### Cancer detection rate stratified by PSA distribution, patient age and PV

The cancer detection rate stratified by PSA distribution is shown in Table 3. The cancer detection rate stratified by PSA value from, 0–4, 4–10, 10–20, 20–30, 30–40, 40–50, 50–10, and more than 100 ng/mL was 20.7, 37.6, 58.4, 80.6, 87.5, 100, 93.3, and 100 %, respectively. The cancer detection rate gradually increased according to the increase in the PSA value.

**Table 3:** Cancer detection rates according to PSA stratification

PSA (ng/mL)	Number of biopsies		Cancer	
	n = 936	%	n = 449	%
0–4	87	9.3	18	20.7
4–10	548	58.5	206	37.6
10–20	154	16.5	90	58.4
20–30	36	3.8	26	80.6
30–40	24	2.6	21	87.5
40–50	10	1.1	10	100.0
50–100	30	3.2	28	93.3
100<	47	5.0	47	100.0

The cancer detection rate stratified by patient age and PV in all patients is shown in Table 4. The cancer detection rate stratified by patient age of ≤59, 60–64, 65–69, 70–74, 75–79, and ≥80 was 25.8, 39.8, 39.8, 46.4, 59.8, and 68.0 %, respectively. The cancer detection rate in older patients was higher than that in younger patients (p < 0.001). The cancer detection rate stratified by PV of ≤25, 25.1–50, and ≥50.1 were 29.9, 43.8, and 66.1 %, respectively. Older patients and those with a small PV showed higher cancer detection rates (p < 0.001). These trends also could be seen in for the gray zone PSA levels (4.1–10.0 ng/mL) (p < 0.001) (Table 5). The cancer detection rate in the gray zone was 37.6 %. The cancer detection rate in the gray zone stratified by patient age of ≤59, 60–64, 65–69, 70–74, 75–79, and ≥80 years were 28.4, 35.0, 26.9, 37.9, 45.7, and 54.8 %, respectively. The cancer detection rate in the gray zone stratified by PV of ≤25, 25.1–50, and ≥50.1 was 20.4, 31.3, and 58.1 %, respectively.

**Table 4:** Cancer detection rates stratified by age and prostate volume in all patients

PV (mL)	Age						
	−59	60–64	65–69	70–74	75–79	80−	Total
−25	32.4 (11/34)	75.8 (25/33)	52.5 (31/59)	66.7 (42/63)	79.7 (47/59)	83.0 (39/47)	66.1 (195/295)
25.1–50	19.6 (9/46)	25.6 (11/43)	43.0 (37/86)	45.4 (59/130)	55.1 (49/89)	58.5 (31/53)	43.8 (196/447)
50.1−	30.8 (4/13)	13.6 (3/22)	14.6 (6/41)	21.7 (10/46)	42.6 (20/47)	60.0 (15/25)	29.9 (58/194)
Total	25.8 (24/93)	39.8 (39/98)	39.8 (74/186)	46.4 (111/239)	59.5 (116/195)	68.0 (85/125)	48.0 (449/936)

**Table 5:** Cancer detection rate stratified by age and prostate volume in gray zone patients

PV (mL)	Age						
	−59	60–64	65–69	70–74	75–79	80−	Total
−25	41.7 (10/24)	71.4 (15/21)	38.2 (13/34)	63.6 (28/44)	66.7 (20/30)	73.7 (14/19)	58.1 (100/172)
25.1–50	20.0 (7/35)	18.5 (5/27)	27.9 (12/43)	32.2 (28/87)	38.6 (17/44)	46.9 (15/32)	31.3 (84/268)
50.1−	25.0 (2/8)	8.3 (1/12)	11.1 (3/27)	16.7 (5/30)	30.0 (6/20)	45.5 (5/11)	20.4 (22/108)
Total	28.4 (19/67)	35.0 (21/60)	26.9 (28/104)	37.9 (61/161)	45.7 (43/94)	54.8 (34/62)	37.6 (206/548)

The total number of biopsy cores was 8420 in this study. Approximately, 25 % of biopsy cores could be reduced using the NURTG nomogram compared with 12-cores biopsy (8420 cores vs. 11,232 cores).

The significant predictive parameters of cancer detection in the gray zone were age, VBR, PSAD, DRE findings, and TRUS findings in univariate analyses. Finally, age, VBR, and PSAD were independent parameters that predicted cancer detection in the gray zone (Table 6).

**Table 6:** Logistic regression analysis predicting positive biopsy in the gray zone

Variables	Univariate		Multivariate		
	p value	Odds ratio	p value	Odds ratio	95 % CI
Age	0.005	1.038	0.001	1.053	1.022–1.084
VBR (median 3.83)	<0.001	0.375	0.008	0.468	0.267–0.818
PSAD (median 0.17)	<0.001	3.924	0.001	2.556	1.506–4.338
DRE	0.003	2.295	0.468	1.280	0.657–2.495
TRUS	0.002	2.232	0.081	1.690	0.937–3.048

*C.I*. confidence interval

The profile of adverse events of grade 2 or greater related with prostate biopsy are shown in Table 7. One patient (0.1 %) suffered from grade 3 prostate infection. 10 patients (0.5 %) showed acute grade 2 urinary retention. We experienced five patients (0.5 %) with hematuria (grade 2), five patients (0.5 %) with fever (grade 2), and two patients with rectal hemorrhage (0.2 %).

**Table 7:** Incidence rate of adverse event ≥ grade 2 after prostate biopsy (CTCAE ver. 4.0)

	Grade 2	Grade 3
Hematuria	0.5 (5)	
Rectal hemorrhage	0.2 (2)	
Prostate infection	0.5 (5)	0.1 (1)
Acute urinary retention	1.1 (10)	
Fever	0.5 (5)	

## Discussion

In our previous retrospective studies on prostate biopsy [6, 7], we used 6–8 biopsy cores, and the cancer detection rate in all patients and patients with gray zone PSA was relatively low. Therefore, we conducted the present prospective study to elucidate the usefulness of the NURTG nomogram allocating 6–12 biopsy cores, the number generally used in daily practice, based on age and PV. Consequently, the cancer detection rate in all patients and the cancer detection rate in gray zone PSA patients increased to 48.0 and 37.6 %, respectively.

Since Hodge introduced systematic sextant biopsy [1], the number of biopsy cores has increased to achieve a higher cancer detection rate [8–11]. For the initial diagnosis, a core biopsy of 10–12 systematic transrectal or transperineal peripheral zone biopsies under ultrasound imaging guidance is recommended by the guideline of the European Association of Urology [12]. Increasing the number of cores can expectedly lead to a higher cancer detection rate. For example, Kawakami et al. introduced a 3-D biopsy method with 26 cores. The cancer detection rate in the gray zone was 36.0 % [13]. Remizi et al. reported the Vienna nomogram in which a number of 6–18 biopsy cores based on PV and age was set. The cancer detection rate was 36.7 % at a PSA level between 2–10 ng/mL [2]. Is it indeed necessary to obtain such a large number of cores for the initial biopsy?

We conducted the present study to address the issue of the optimal number of cores in daily practice. We regulated the number of cores from 6 to 12 based on patient age and PV. Younger patients and a larger PV were allocated to more biopsy cores. This concept was similar to that of the Vienna nomogram [2]. The cancer detection rate in the gray zone of the present study was 37.6 % (cf. Vienna nomogram 36.7 %). Our present results revealed that the NURTG nomogram, which regulated the number of cores at 6–12 based on age and PV, achieved similar cancer detection rates as previous studies conducted in larger cohorts (Table 8) [2, 13–15]. If all patients received 12-cores biopsy in this study cohort, the total number of biopsy cores was 11,232. In fact, the total number of biopsy cores was 8420. Approximately, 2812 cores (25 %) could be reduced using the NURTG nomogram, and a huge decrease in pathology time and costs could be obtained. A small prostate is also an important and noteworthy sign of prostate cancer detection. Eventually, multivariate analysis showed that PSAD, VBR and age were independent parameters that predict prostate cancer in the gray zone (Table 6). Jiang et al. first reported that the cut-off value of VBR ≤4 was optimal for detecting cancer and avoiding excessive biopsy specimens in patients with PSA values of <20 ng/mL [5]. Miyoshi et al. also confirmed that VBR was an independent parameter to predict prostate cancer in patients with PSA values of 4–20 ng/mL [16]. Our present results also confirmed that VBR (cut-off value 3.83) was an independent predictor of prostate cancer for gray zone PSA levels (4.1–10.0 ng/mL). We should interpret the meaning of VBR carefully. The fact that the cancer detection rate is higher in patients with a smaller VBR means that the number of biopsy cores is adequate and that patients with a smaller PV are more likely to have prostate cancer. This result is also confirmed by the fact that patients with a higher PSAD show a higher cancer detection rate. In the present study, PSAD was also an independent predictor of prostate cancer detection like age and VBR (Table 6). Younger patients with a smaller PV also showed a lower cancer detection rate compared with older patients, independent of whether younger patients underwent biopsy with a greater number of cores (Tables 4, 5). The conceivable reason for these results is that the cancer volume of younger patients is probably smaller than that of older patients.

**Table 8:** Comparison of cancer detection rates in previous reports of studies in large numbers of patients

Reference	No of pts	No of cores	TZ biopsy	PSA range	Cancer detection rate (%)	
					All cases	Gray zone
Ung [14]	750	12 (6–18)	NA	0.3–67.0 Median 4.4	33.7	NA
Remzi [2]	502 Vienna nomogram	6–18 (mean 10)	NA	2–10		36.7
Kawakami [13]	663	26 (TP16 + TR12)	+	<20	36	36 (4–939)
Takenaka [15]	247 repeat 58	12 (TP)	+	4–100	39.7	34.4 (4–10)
Present study	936 NURTG nomogram	6–12	NA	0.3–16.920 Median 7.1	48.0	37.6

*TZ* transition zone, *TP* transperineal biopsy, *TR* transrectal biopsy, *NA* not available

The present study showed an acceptable result of adverse events after biopsy (Table 7). The incidence of adverse events ≥grade 2 after prostate biopsy was acceptable compared with previous studies [17]. We believe that our method using NURTG nomogram is safe and reliable in respect of adverse events. The cancer detection rate in our study is also equivalent to that in previous studies with a greater number of biopsy cores (Table 8). The number of biopsy cores can be reduced in patients of ≥70 years of age instead of the uniform 12-core biopsy.

## Conclusion

Our present study revealed that the cancer detection rate using the NURTG nomogram allocating a number of 6–12 biopsy cores, the number generally used in daily practice, based on age and PV could lead to similar efficacy compared with previous studies with a more expanded number of biopsy cores. In older patients the number of biopsy cores can be reduced.

## Abbreviations

PSA: prostate specific antigen
NURTG: Nara Urological Research and Treatment Group
TRUS: transrectal ultrasound
PV: prostate volume
VBR: volume biopsy ratio
PSAD: PSA density
DRE: digital rectal examination
